# The impact of an educational program on the electronic waste management knowledge and practices of dental interns: an interventional study

**DOI:** 10.1038/s41598-026-46718-0

**Published:** 2026-04-13

**Authors:** Rana Samy Galal, Aleya Hanafy El-Zoka, Ebtisam Mohamed Fetohy, Mayada Mohamed Reda Moussa, Mohamed Fakhry Hussein

**Affiliations:** 1 Department of Conservative Dentistry, Faculty of Dentistry, Alexandria University, Alexandria, 21571 Egypt; 2 Department of Occupational Health and Industrial Medicine, High Institute of Public Health, Alexandria University, Alexandria, 21561 Egypt; 3 Department of Health Administration and Behavioral Sciences, High Institute of Public Health, Alexandria University, Alexandria, 21561 Egypt

**Keywords:** Dentistry, Educational programs, Electronic waste, Environment, Knowledge, Practice, Diseases, Health care, Medical research

## Abstract

The increasing volume of electronic waste (e-waste) poses considerable risks to the environment and humans. This study evaluated an educational program impact on e-waste management among dental interns at Faculty of Dentistry, Alexandria University, Egypt. This interventional quasi-experimental study without control group used a validated self-administered questionnaire to assess participants’ awareness, knowledge, and reported practices before, immediately after, and three months following the educational intervention on 76 participants. Data was analyzed using the Friedman test with Dunn’s post-hoc comparisons and Spearman’s correlation. At baseline, awareness, knowledge, and practice mean score percentages (15.45%±5.26%, 16.07%±13.81%, 17.94%±14.84%, respectively) were low. Following the intervention, significant increases were observed across all domains at both the immediate and three-month intervals (*p* < 0.001). While awareness and knowledge scores peaked immediately and showed slight decline at three months, self-reported practice mean score percentages demonstrated a progressive upward trend, reaching 72.13%±17.08% at the final follow-up. Significant positive correlations between knowledge and practice before and after the program were observed (*p* < 0.05). The present program was associated with improvements in participants’ awareness, knowledge, and practices regarding e-waste management; however, these findings should be interpreted cautiously due to the pre–post design, reliance on self-reporting and absence of a control group.

**Supplementary Information:**

The online version contains supplementary material available at 10.1038/s41598-026-46718-0.

## Introduction

Electronic waste (e-waste), comprising discarded electronic products nearing or beyond their useful life, poses a critical environmental challenge. Improperly managed e-waste, often disposed of in landfills or dumping sites, releases hazardous substances such as lead, mercury, cadmium, and polycyclic aromatic hydrocarbons into soil, water, and air, disrupting ecosystems and endangering biodiversity. These detrimental effects can persist for generations^1–4^. Human exposure to these pollutants can lead to a range of health problems, including respiratory problems, neurological disorders, kidney and liver insufficiency, reproductive impairments, and even cancer^5^.

Egypt is one of the top African countries in electronic consumption, which has led to the increase in the amount of e-waste generated^6^. The average e-waste generated in Egypt was about 20% of that generated in Africa^7^.

Dentists rely heavily on electrical and electronic equipment (EEE) to deliver modern dental care. The constant drive for technological advancements leads to frequent equipment upgrades, contributing significantly to the growing volume of e-waste in the dental sector. Common EEE in dental practice includes amalgamators, apex locators, x-ray machines, curing lights, handpieces, dental chairs, endodontic tools, ultrasonic scalers, and intraoral cameras^8,9^. As dentists are responsible members of society, they should be aware of the hazardous impacts of e-waste generation, its prevention, and proper management^10^.

Dental interns occupy a critical stage in this professional landscape; as they transition from institutional learning to independent practice, they establish the waste management habits that will define their future clinical environmental footprint. However, a significant gap exists between the increasing reliance on digital technology and the formal training provided on its sustainable disposal particularly, in low- and middle-income countries such as Egypt. Without targeted educational interventions, this distinct population remains unprepared to manage the environmental and regulatory challenges of digital dentistry, leading to improper disposal practices that contribute to the broader e-waste crisis. This gap highlights the need to assess and improve e-waste-related knowledge and practices among this group^11–13^.

To minimize the detrimental impact of electronic waste on public health and the environment, effective e-waste management through the Reuse, Recycle, Reduce, and Repurpose (4R) framework is crucial. Adopting this approach can significantly raise consumer awareness and foster sustainable practices for proper e-waste disposal^14^.

Environmental educational programs are highly effective tools for enhancing environmental knowledge and promoting responsible practices^15–17^. In 2021, a study by Vibhute et al. in India demonstrated that the educational program was effective in enhancing dental students’ knowledge of e-waste management. Such initiatives are critical to improving the handling of waste electrical and electronic equipment (WEEE) by improving consumer awareness and promoting responsible disposal practices^10^. We hypothesized that an educational program would significantly improve dental interns’ knowledge and practices regarding e-waste management, addressing the current awareness gap in this region.

## Methods

### Aim of the study

The present study aimed at evaluating dental interns’ awareness, knowledge, and practices regarding e-waste management and assessed the effectiveness of the educational intervention through pre- and post-intervention comparisons.

### Study design and setting

It was an interventional quasi-experimental one-group pre-test post-test study, which was conducted at the Faculty of Dentistry, Alexandria University, Egypt, during the period from July to December 2024. This study was conducted as a pilot interventional trial at a single major institution to establish baseline evidence and evaluate the feasibility of the educational module before wider implementation. This institution is one of the largest dental training centers in the region, with interns coming from various educational backgrounds, which may reflect the standardized national curriculum.

### Study population

The target population was the intern dentists who were practicing at Faculty of Dentistry, Alexandria University, Egypt, during the period of the study.

### Sampling method and sample size

**Fig. 1:**
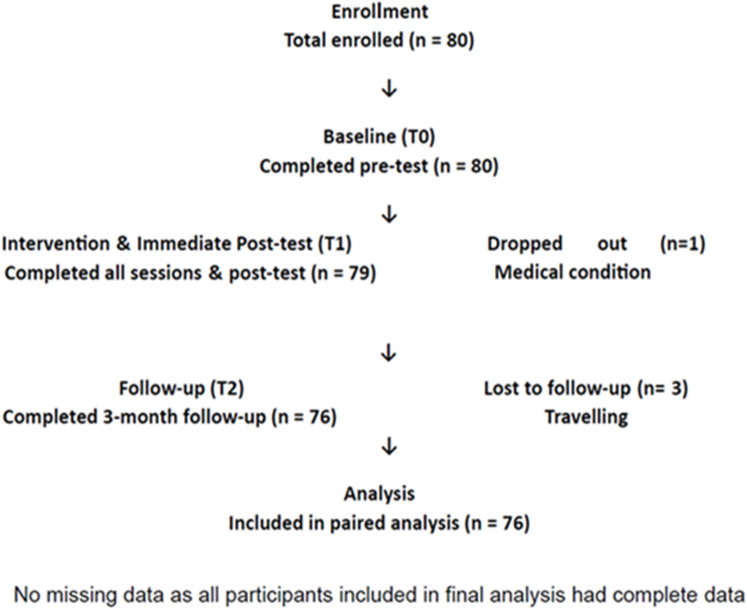
A flowchart detailing the recruitment and retention process.

Based on the assumption that the effect size of the difference in knowledge and practices of intern dentists regarding e-waste management before and after the educational program was 0.35. Using an alpha error of 0.05 and 80% power, the minimum sample size required was 67 subjects. Considering a 10% dropout ratio or attrition, the sample size was increased to 74^10^. The sample size was calculated using G-Power software. A total population sampling approach was employed, which included all intern dentists (*n* = 80) practicing at the Faculty of Dentistry during the study period. While this constitutes a convenience sample relative to the national population, it ensured a comprehensive assessment of the specific institutional cohort transitioning into professional practice. During the study, one participant was lost at the immediate post-intervention phase due to a medical condition, and three others were lost at the three-month follow-up due to travel. Despite these losses, the final longitudinal cohort consisted of 76 participants (*n* = 76), which remained above the power-calculation threshold. Consequently, the study’s statistical validity and its ability to detect significant changes were not compromised. To ensure data integrity, quality checks were conducted immediately after each assessment stage to verify completeness and accuracy before the participants left the study site. If incomplete entries were identified, participants were immediately requested to provide the missing information. Any record that remained incomplete across the three longitudinal time points was excluded from the final analysis. The final analysis only included participants with complete data across all three time points, thereby maintaining the study’s longitudinal validity. The recruitment and retention process is detailed in the flowchart in Fig. 1.

### The data collection tool

The research was carried out in the following three stages:

### Stage I: A pre-intervention phase

#### A predesigned self-administered questionnaire (Supplementary Material I)

A pre-designed, pre-coded, self-administered questionnaire was developed. It consists of 30 questions to collect data from participants following an extensive literature review^3,10,18^ and has undergone a structured construct and content validation process. To ensure content validity, a draft was reviewed by a panel of three experts in dental health, public health, and environmental science; items were refined based on their feedback to ensure technical accuracy and relevance. Subsequently, the instrument was pilot-tested on a representative group of 10 dental interns to assess the clarity of the questions. The survey instrument aimed to assess dental interns’ awareness, knowledge, and practices regarding e-waste management. Construct stability was further verified by calculating the internal consistency of awareness, knowledge, and practices scales, yielding a Cronbach’s alpha coefficient of 0.655 for the awareness part, which was acceptable for exploratory educational research; 0.879 for the knowledge part; and 0.842 for the practice part. These results indicated an acceptable level of reliability^19,20^. To mitigate social desirability bias, which is the tendency of participants to provide “ideal” rather than “honest” answers, the questionnaires were administered anonymously. Furthermore, the “Knowledge” and “Awareness” sections consisted of objective, fact-based questions (e.g., identifying specific hazardous components of e-waste or existing legislation) rather than purely subjective or attitudinal questions. This makes it more difficult for participants to “guess” the correct answer based on social expectations alone and to show the effect of the educational intervention afterwards.

The questionnaire consisted of four main parts: The first part was concerned with personal information such as sex, age, marital status, number of family members, their participation in any previous training program for e-waste management, and the source of their information. The second part was related to the intern dentists’ awareness about e-waste management. It included questions related to whether they heard about the e-waste terminologies, the E-tadweer application, and their awareness about e-waste recycling. Each of these questions had a score of “1” for a “Yes” answer and a score of “0” for a “No” answer. Total awareness scores were calculated, converted into percentages, and categorized as good (> 75%), fair (50–75%), and poor (< 50%).

The third section assessed dentists’ knowledge of e-waste management. Participants responded to statements and questions about e-waste definition, environmental and health impacts, disposal methods, recycling, and knowledge of governmental regulations. Topics also included the concept of the 4R, the presence of valuable components in e-waste, and appropriate disposal sites. Responses were categorized as “yes,” “no,” or “do not know,” with correct answers assigned a score of 1 and incorrect or “don’t know” responses receiving a score of 0.

The final knowledge question asked participants to identify hazardous materials present in e-waste. Responses were scored on a scale of 0 to 2, with 2 points awarded for correctly identifying three or more materials, 1 point for one or two correct answers, and 0 points for incorrect or unknown responses. Total knowledge scores were calculated, converted into percentages, and categorized as good knowledge (> 75%), fair knowledge (50–75%), and poor knowledge (< 50%).

The last part was about self-assessment of dental practices regarding e-waste. Participants were asked about commonly used EEE in their personal and professional lives, reasons for purchasing new devices, and their disposal methods for outdated electronics. Regarding their practices for electronics that are no longer in use, their responses were scored on a scale of 0 to 3, with higher scores indicating more environmentally responsible actions, such as donating to e-waste collectors. Lower scores reflected improper disposal, including discarding in trash or landfills. While the last four questions were concerned about their practices regarding the separation of WEE from general waste, encouraging others to recycle e-waste, implementing recycling within their families, and purchasing eco-friendly products. Responses were rated on a scale of 0 to 2, with a score of “2” for always response, a score of “1” for sometimes response, a score of “0” for never response. After calculating and converting the total practice scores into percentages, the results were divided into three categories: good practice (> 75%), fair practice (50–75%), and poor practice (< 50%).

### Pilot study

A pilot study involving 10 intern dentists who were practicing in a previous shift at the Faculty of Dentistry was conducted to refine the study questionnaire and assess data collection feasibility (not included in the study sample). Objectives included evaluating the clarity of the questionnaire, confirming its reliability, estimating the time required for completion (15 to 20 min), and identifying any potential obstacles during data collection. Based on pilot feedback, minor modifications were made to the questionnaire for improved clarity and significant obstacles were encountered.

### Stage II: Intervention educational program

An intervention program was designed to enhance intern dentists’ knowledge and practices regarding e-waste management. The 76 participants were divided into four groups of 19. Each group received four educational sessions (one session per week) over the period of one month to ensure adequate coverage of key topics while remaining feasible within the internship schedule. These hour-long sessions combined lectures with audiovisual aids (PowerPoint presentations, images, and videos) and interactive group discussions. Printed handouts and posters complemented the learning experience. The intervention consisted of four sessions to align with the principle of Spaced Learning^21,22^, which suggests that distributed sessions enhance long-term retention compared to massed learning. Besides, the divided content guarantees the avoidance of cognitive overload among dental interns who have demanding clinical schedules. So, the content was divided into four shorter, manageable sessions rather than one long seminar. Each session focused on a specific thematic pillar of e-waste management to ensure comprehensive coverage while minimizing cognitive fatigue among the participants. The educational program covered the following topics: foundational knowledge of e-waste, including definitions, types, global burden, and specific dental e-waste; the hazards associated with e-waste, detailing the toxic chemicals involved and their adverse impacts on human health and the environment; practical e-waste management strategies, encompassing the benefits of recycling, government responsibilities, current regulations, proper disposal protocols, and designated collection points; and finally, the implementation of the 4R principles (Reduce, Reuse, Recycle, Repurpose) in dental practice and the importance of sustainable procurement of dental devices. A key practical component of the training involved the use of the E-tadweer application, a national digital platform launched by the Egyptian Ministry of Environment. This application facilitates the regulated collection of e-waste by connecting users with certified recycling points. Participants were instructed on the app’s interface, which includes waste categorization, photo uploading, and a reward system involving discount vouchers. Interns were trained on how to use the app specifically for dental-related e-waste (such as sensors, old curing lights, or office electronics). This provided the interns with a tangible mechanism for applying their theoretical knowledge to real-world dental practice.

### Stage III: Post-intervention phase

Re-evaluation of knowledge and practice of study group members was carried out by recollection of data immediately at the end of the last educational sessions and three months later using the same questionnaire. The program effectiveness was evaluated by comparing participants’ assessments before the program, immediately after, and three months later. Participant confidentiality was maintained using a self-generated anonymized coding system. At each phase, participants provided the same unique code, allowing researchers to link responses across the three time points without collecting identifiable data. This linkage facilitated the use of paired/dependent statistical analyses (Friedman’s and Spearman’s tests), ensuring a more accurate assessment of the intervention’s impact over time.

### Ethical considerations

The study protocol was reviewed and approved by the Ethics Committee of the High Institute of Public Health, Alexandria University, Egypt, with IRB number: 00013692, prior to the start of the study in July 2024. Approval from the dean of the Faculty of Dentistry, Alexandria University, was taken. The researchers adhered to the ethical principles outlined in the Declaration of Helsinki. The study was registered with the Pan African Clinical Trial Registry (The clinical trial registration number: PACTR202408837898812, Date: 20 August 2024, (Supplementary Material II). While registration was sought before the beginning of the study, technical and administrative delays resulted in the final approval being granted in August, classifying it as “retrospectively registered”. This timing did not impact study integrity; all interventions and outcome measures were executed in strict adherence to the IRB-approved protocol, with no post-hoc changes or deviations made to the methodology. Informed consent was taken from all participants in the study after an explanation of the purpose and benefits of the research. Anonymity and confidentiality were assured and maintained. Any participant could choose to leave the study at any time before it ended.

### Statistical analysis

Data was fed to the computer and analyzed using IBM SPSS software package version 20.0 **(**Armonk, NY: IBM Corp.). Qualitative data were described using number and percentage. The Kolmogorov-Smirnov Wilk test was used to verify the normality of distribution. Quantitative data were described using range (minimum and maximum), mean, standard deviation (SD), median and interquartile range (IQR). The significance of the results was judged at the 5% level. The Friedman test was used for abnormally distributed quantitative variables to compare more than two periods or stages, and the Post Hoc test (Dunn’s) was used for pairwise comparisons. The Spearman coefficient test (r_s_) was used to test the correlation between two abnormally distributed quantitative variables. Reliability of the tool was assessed using the Cronbach’s Alpha test.

## Results

### Sociodemographic characteristics of the participants

Table 1 presents the participants’ socio-demographic characteristics. Of the 76 participants, 80.3% were female, with a mean age of 23.96 ± 0.70 years. The majority (85.5%) were single, and family sizes ranged from 1 to 8 members. Notably, none had prior e-waste management program experience, and 86.9% were unfamiliar with the concept.

**Table 1:** The Socio-demographic characteristics of the studied participants (*n* = 76).

Personal Information	No.	%
Sex		
Male	15	19.7
Female	61	80.3
**Age**		
23	19	25.0
24	42	55.3
25	14	18.4
26	1	1.3
Min. – Max.	23.0–26.0	
Mean ± SD	23.96 ± 0.70	
**Marital status**		
Single	65	85.5
Married	11	14.5
**Number of family members**		
Min – Max	1.0–8.0	
Mean ± SD	4.26 ± 1.31	
**Participation in previous e-waste management programs**		
No	76	100.0
Yes	0	0.0
**Source of information** ^**#**^ No Social media Friends or family members Television	67 9 1 1	86.9 11.5 1.3 1.3

# Multiple responses SD: Standard deviation.

### The overall awareness of the studied participants

Table 2 summarizes the significant shift in awareness levels regarding e-waste management following the intervention. At baseline, none of the participants demonstrated ‘good’ awareness; however, this improved dramatically in both post-intervention assessments (*p* < 0.001). Notably, mean awareness scores surged from a baseline of 15.45% to over 95% in subsequent evaluations, with these gains showing no significant decline between the immediate and three-month follow-up periods (*p* > 0.05). For more details about the responses, see Supplementary Material III, Table S1.

**Table 2:** The overall awareness of the studied participants (*n* = 76).

Awareness	Pre-intervention		Immediate post-intervention		3 months post-intervention		Significance between periods		
							*p*_1_	*p*_2_	*p*_3_
**Awareness level** Poor (< 50%)	73	96.1	1	1.3	4	5.3	< 0.001^*^	< 0.001^*^	0.656
Fair (50–75%)	3	3.9	1	1.3	2	2.6			
Good (> 75%)	0	0.0	74	97.4	70	92.1			
**Total score (0–3)**									
Mean ± SD	0.16 ± 0.46		2.96 ± 0.26		2.87 ± 0.47		< 0.001^*^	< 0.001^*^	0.715
Median (IQR)	0.0 (0.0–0.0)		3.0 (3.0–3.0)		3.0 (3.0–3.0)				
**Total score percentage**									
Mean ± SD	15.45 ± 5.26		98.68 ± 8.50		95.61 ± 15.72				
Median (IQR)	0.0 (0.0 − 0.0)		100.0 (100.0–100.0)		100.0 (100.0–100.0)				

IQR: Inter Quartile Range SD: Standard deviation. Friedman test, Significance between were done using Post Hoc Test (Dunn’s test). *p*1: *p* value for comparing between pre−intervention and immediate post−intervention. *p*2: *p* value for comparing between pre−intervention and three months post−intervention. *p*3: *p* value for comparing between immediate and three months post−intervention. *: Statistically significant at *p* < 0.05.

### The overall knowledge score of the studied participants

Table 3 illustrates a substantial enhancement in participants’ knowledge levels following the intervention. While baseline knowledge was universally poor, the program yielded a marked increase in both categorical proficiency and mean scores immediately post-intervention (*p* < 0.001). At the three-month follow-up, a statistically significant decrease in knowledge was observed compared to the immediate post-test results (*p* = 0.004); however, scores remained significantly higher than baseline levels (*p* < 0.001). These findings suggest strong initial knowledge acquisition with a partial, yet expected, decrease over the medium term. For more details about the responses, see Supplementary Material III, Table S2.

**Table 3:** The overall knowledge score of the studied participants (*n* = 76).

Knowledge	Pre-intervention		Immediate post-intervention		3 months post-intervention		Significance between periods		
	No.	%	No.	%	No.	%	*p*_1_	*p*_2_	*p*_3_
**Knowledge level** Poor (< 50%)	73	96.1	0	0.0	1	1.3	< 0.001^*^	< 0.001^*^	0.047^*^
Fair (50–75%)	3	3.9	7	9.2	30	39.5			
Good (> 75%)	0	0.0	69	90.8	45	59.2			
**Total score (0–14)**									
Mean ± SD	2.25 ± 1.93		12.34 ± 1.36		10.96 ± 1.89		< 0.001^*^	< 0.001^*^	0.004^*^
Median (IQR)	2.0 (0.50–3.0)		13.0 (12.0–13.0)		11.0 (10.0–12.0)				
**Total score percentage**									
Mean ± SD	16.07 ± 13.81		88.16 ± 9.73		78.29 ± 13.47				
Median (IQR)	14.29 (3.57–21.43)		92.86 (85.71–92.86)		78.57 (71.43–85.71)				

IQR: Inter Quartile Range SD: Standard deviation. Friedman test, Significance between periods were done using Post Hoc Test (Dunn’s test). *p*1: *p* value for comparing between pre−intervention and immediate post−intervention. *p*2: *p* value for comparing between pre−intervention and three months post−intervention. *p*3: *p* value for comparing between immediate and three months post−intervention. *: Statistically significant at *p* < 0.05.

### The overall practice of the studied participants

Regarding the reasons for purchasing new gadgets, the majority cited loss of function (48.7% in pre-intervention, 57.9% in three-month post-intervention), the desire for newer technology (25% in pre-intervention, 19.7% after three months of intervention), and the need for greater functionality (26.3% in pre-intervention, 18.4% after three months of intervention) (Supplementary Material III, Figure S1).

Regarding the practice towards electronics waste, Table 4 highlights a significant transformation in e-waste disposal practices following the intervention (*p* < 0.001). Before the program, the majority of participants kept devices at home, or threw them in the trash, and only 6.6% delivered them to an e-waste collector. Post-intervention, the unsustainable behaviors decreased significantly in favor of delivering e-waste to certified collectors (*p* < 0.001). Notably, these improved disposal habits remained stable between the immediate and three-month assessments (*p* = 0.224), indicating that the program initiated a sustained shift toward environmentally responsible disposal.

**Table 4:** The practice towards electronics that were no longer in use among the studied intern dentist (*n* = 76).

Practice regarding electronics that are no longer in use	Pre-intervention		Immediate post-intervention		3 months post-intervention		Significance between periods		
	No.	%	No.	%	No.	%	*p*_1_	*p*_2_	*p*_3_
(0) Thrown in trash/landfills - Burning/ incineration	13	17.1	7	9.2	4	5.3	< 0.001^*^	< 0.001^*^	0.224
(1) Kept at home	41	53.9	23	30.2	18	23.6			
(2) Given to personal contact - Exchanged with dealer	17	22.4	11	14.5	16	21.1			
(3) Given to e-waste collector	5	6.6	35	46.1	38	50.0			

Friedman test, Significance between periods were done using Post Hoc Test (Dunn’s test). *p*1: *p* value for comparing between pre−intervention and immediate post−intervention. *p*2: *p* value for comparing between pre−intervention and three months post−intervention. *p*3: *p* value for comparing between immediate and three months post−intervention. *: Statistically significant at *p* < 0.05.

Table 5 details the intern dentists’ overall e-waste management practices before and after the educational program. A progressive and significant improvement in overall e-waste management practices over the course of the study was observed (*p* < 0.001). While no participants demonstrated ‘good’ practice at baseline, there was a steady upward trajectory in both categorical proficiency and mean practice scores throughout the post-intervention period. The mean practice score percentage also showed a significant increase, rising from 17.94% pre-intervention to 49.28% immediately post-intervention and further to 72.13% at the three-month follow-up (*p* < 0.001). For more details about the responses, see Supplementary Material III, Table S3.

**Table 5:** The overall practice of the studied participants (*n* = 76).

Practice	Pre-intervention		Immediate post-intervention		3 months post-intervention		Significance between periods		
	No.	%	No.	%	No.	%	*p*_1_	*p*_2_	*p*_3_
**Practice Level** Poor (< 50%)	73	96.1	43	56.6	8	10.5	0.001^*^	< 0.001^*^	< 0.001^*^
Fair (50–75%)	3	3.9	24	31.6	38	50.0			
Good (> 75%)	0	0.0	9	11.8	30	39.5			
**Total score (0–11)**									
Mean ± SD	1.97 ± 1.63		5.42 ± 2.67		7.93 ± 1.88		< 0.001^*^	< 0.001^*^	< 0.001^*^
Median (IQR)	2.0 (1.0–3.0)		5.0 (4.0–7.0)		8.0 (7.0–9.0)				
**Total score percentage**									
Mean ± SD	17.94 ± 14.84		49.28 ± 24.32		72.13 ± 17.08				
Median (IQR)	18.18 (9.09–27.27)		45.45(36.36–63.64)		72.73(63.64–81.82)				

IQR: Inter Quartile Range SD: Standard deviation. Friedman test, Significance between periods were done using Post Hoc Test (Dunn’s test). *p*1: *p* value for comparing between pre−intervention and immediate post−intervention. *p*2: *p* value for comparing between pre−intervention and three months post−intervention. *p*3: *p* value for comparing between immediate and three months post−intervention. *: Statistically significant at *p* < 0.05.

### The correlation between knowledge and practice scores

**Fig. 2:**
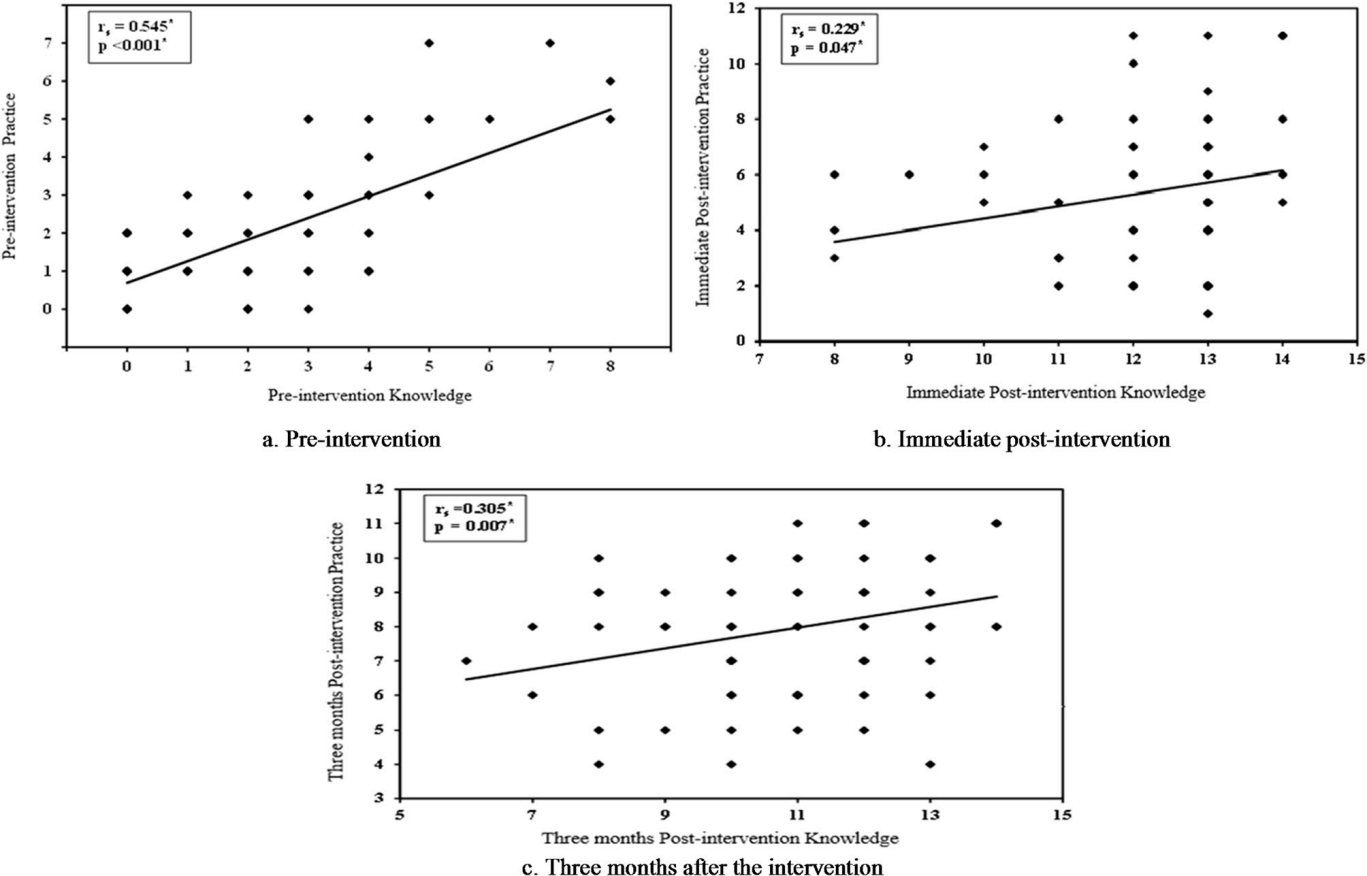
The correlation between knowledge and practice scores.

As illustrated in Fig. 2 (a, b, and c), a statistically significant positive correlation was found between participants’ knowledge and practice of e-waste management at pre-intervention (*p* < 0.001), immediately post-intervention (*p* = 0.047), and three months post-intervention (*p* = 0.007), which indicated that increased knowledge was associated with improved e-waste management practices.

## Discussion

In the present study, we implemented an educational program on e-waste management and evaluated its effectiveness among junior dental staff. The exceptionally high immediate post-intervention awareness scores demonstrate the efficacy of the intensive four-week module in bridging a baseline awareness gap that was previously near zero. While these scores reached a near-ceiling level—potentially limiting the questionnaire’s ability to discriminate between high-achieving participants—this was largely because the awareness assessment focused on fundamental knowledge rather than higher-order application. However, this effect was attenuated during the three-month follow-up, allowing more meaningful analysis of long-term awareness stability. Besides, the three-month interval between assessments, coupled with the subsequent decline in follow-up scores, indicates that the initial gains were not solely due to the testing effect; rather, they reflected genuine learning instead of mere familiarity with the questionnaire^23,24^.

The significant improvement in e-waste awareness among dental interns following our educational program aligns with findings by Subhaprada et al.^25^, whose study on medical students also demonstrated that targeted interventions can successfully bridge initial awareness gaps. These findings shed light on the importance of targeted educational programs in raising awareness about e-waste. However, our baseline findings contrast with Cheruvalappil et al.^26^, who reported higher initial awareness in their cohort, with 44% poor, 27.1% fair, and 28.9% good awareness among participants. This discrepancy likely stems from methodological differences: while our study focused on a specific, newly graduated intern population in Egypt with no prior exposure to e-waste education, the broader population sampling in northern Kerala may have captured individuals with more diverse informal exposure to environmental topics. A cross-sectional study carried out by Yee et al. in Malaysia also found a higher awareness level about e-waste than ours, as 27.4% of medical students showed good awareness, which may be due to previous exposure to this issue in their curriculum, yet this awareness was still insufficient^27^.

In the present study, the initial lack of knowledge regarding e-waste hazards mirrors the findings of Nuwematsiko et al.^28^ in Uganda, where 67.7% of participants had poor e-waste management knowledge, and a cross-sectional study in India where only 3.2% of dental students had good knowledge^3^. Although this study lacked a control group, the severity of the baseline ‘knowledge gap’ suggests that the observed improvements were unlikely to occur spontaneously without the intervention. To validate these gains, we utilized a longitudinal approach with a three-month follow-up. While participants showed a slight decline in knowledge retention at the three-month mark, scores remained substantially higher than baseline. This pattern suggests that while intensive four-week modules are effective for immediate theoretical gains, long-term professional retention requires periodic reinforcement rather than a single, isolated intervention.

When participants were asked about reasons for purchasing new gadgets, the majority cited loss of function and desire for new technology as primary reasons, followed by the need for greater technology. In agreement with the present study, a study in China reported that 52% of respondents replace their gadgets due to loss of function^29,^ and a study in India found that 67% of health sciences students would buy new devices when their own devices were damaged^30^. The desire for the newest technology was the leading reason for purchasing new gadgets by dentists in Katti’s study (38%)^31^, while in a Nigerian study, the main reasons for buying new electronic equipment were to replace damaged ones (49.6%) and upgrade their gadgets (37.7%)^32^. These findings indicated that while practical considerations such as device malfunction remain important drivers for gadget replacement, there’s also a notable trend toward seeking out new technologies and enhanced functionality among consumers. Also, the current lifestyle and speed of technology motivate people to get the newest devices, which unfortunately increases the burden on the environment and requires raising public awareness and practices regarding the safe disposal of old, unused gadgets. These findings underline the importance of manufacturing these gadgets from eco-friendly components and considering their life span longevity to decrease their harmful environmental impacts. Besides, it shed light on the importance of the integration of 4R principles (Reduce, Reuse, Recycle, Repurpose) specifically within dental curricula^10,31^.

At baseline, our participants’ self-reported tendency to keep e-waste at home or dispose of it in general trash was remarkably similar to behaviors documented in Malaysia^33^ and India^34^. Similarly, a study in northern Kerala stated that 53.8% of participants stored e-waste at home and 16.9% disposed of it in the trash^26^. Interestingly, while many Indian respondents had access to recycling centers^26,34^, our participants initially lacked knowledge of where to dispose of such waste. The shift in our study toward reporting using e-waste collectors post intervention suggests that when educational programs provide specific, actionable information such as the use of the E-tadweer application they can directly overcome the “lack of knowledge on where to dispose” identified as a barrier in previous descriptive studies.

The significant decrease in the percentage of those who reported keeping unused electronics at home in the current study after the intervention aligns with findings from Borthakur and Singh^35^ and Bandyopadhyay et al.^36^. This may be due to awareness improvement that could significantly influence adoption of disposal practices. The shift from “Keeping e-waste at home or at the clinic” (pre-intervention) to “utilizing e-waste collectors” (post-intervention) represents a tangible reduction in environmental biohazards. By diverting discarded electronics from improper storage or general waste streams, this self-reported behavioral change directly reduces the risk of toxic leaching—such as lead, mercury, and cadmium—into the local ecosystem. Furthermore, utilizing formal collection channels ensures that hazardous components are processed through regulated recovery cycles rather than accumulating as “hidden” biohazards in dental facilities. This shift suggested that targeted education can successfully transform passive storing into active, responsible disposal, thereby mitigating the long-term environmental footprint of digital dentistry. Besides, the sustained improvement in the “self-assessment practice” domain at three months is particularly reflecting changes in behavior over a considerable period, which is less likely to be influenced by simple conditioning from the initial test in the study. Notably, while novelty may have boosted initial engagement in the study, the sustained retention of knowledge and long-term habit formation (observed at 12 weeks) are indicators of successful learning rather than a temporary spike in interest^37,38^.

Most of the participants did not know the appropriate methods for disposing of their electronic garbage as reported at the baseline assessment, which contrasts the results reported by Yee et al. where 67.4% of Malaysian medical students appropriately get rid of electronic gadgets and 41.5% of them properly disposed of electrical appliances^27^. This finding in the present study may explain why people frequently keep e-waste in their homes and dispose of it with other types of trash. One important consideration is that the storage technique was selected more than the disposal or reuse of old electronics. This may be due to a lack of knowledge on where to get rid of e-waste. This highlights the importance of the educational program to increase awareness and knowledge about e-waste management to promote a more responsible practice towards proper e-waste disposal methods^10^.

Finally, the positive correlation between knowledge and self-reported practice noticed in our study supports the findings of Azodo et al.^39^ among Nigerian collegiate students. Unlike cross-sectional surveys that only capture a single point in time, our interventional design demonstrated that even when theoretical knowledge slightly decreased, improved practices can be sustained. This suggests that the intervention helped develop professional habits that may persist regardless of minor fluctuations in factual recall.

The findings of this study have significant clinical implications for the modern dental practice, where the transition to digital workflows is no longer optional but standard. The present study suggested that improved e-waste management knowledge could directly enhance the sustainability and safety protocols of a clinic. Specifically, the integration of 4R principles (Reduce, Reuse, Recycle, and Recover) and the establishment of accessible collection points within dental facilities can mitigate the release of biohazardous electronic components into the ecosystem. To build upon these results and address current limitations, future research should implement longitudinal tracking beyond twelve months to evaluate long-term knowledge retention and the required frequency of refresher training. Furthermore, moving beyond self-reported surveys toward objective behavioral audits, such as direct bin-content analysis, would provide a more precise measurement of actual clinical practice. Finally, conducting economic cost-benefit analyses regarding formal e-waste collection versus conventional disposal will provide the essential evidence needed by policymakers to incentivize and standardize green dentistry practices on a national and international scales.

### Limitation and strength of the study

The present study has some limitations. First, the use of a convenience sample from a single academic institution may introduce selection bias and limit the generalizability of the results to dental practitioners in different geographic or private-sector settings. Future multi-center studies involving diverse clinical environments are needed to validate these findings on a broader scale. Second, the absence of a control group restricts our ability to definitively establish causality or exclude the “testing effect,” where repeated assessments might make participants “test-wise.” Besides, the large gains should be interpreted as a reflection of the significant baseline knowledge gap. Third, the reliance on self-reported data may introduce social desirability and recall biases, although the use of objective knowledge questions and anonymous surveys was intended to mitigate these factors. Fourth, due to the ceiling effect observed in the immediate post-intervention awareness scores, as a high proportion of participants achieved near-maximum scores, the questionnaire’s ability to discriminate between varying levels of awareness at that specific time point was restricted. Furthermore, while the three-month follow-up showed encouraging trends, it may not fully capture the long-term sustainability of behavioral changes beyond the medium term. Finally, while the retrospective trial registration resulted from administrative delays and did not impact the study’s execution, we recognize that prospective registration remains the gold standard for transparency. Collectively, these factors suggest that while the program is a successful pilot, its results should be interpreted as a promising association requiring further validation through randomized controlled trials.

However, this study has several strengths. First, it addresses the under-researched area of e-waste management within dentistry, providing valuable insights into critical environmental and public health issues. Second, the pre-post intervention design, with immediate and three-month follow-up assessments, enabled us to track the medium-term knowledge retention and the potential for meaningful behavioral shifts. Third, this is among the first studies to assess the impact of e-waste management education on junior dental staff. Fourth, the comprehensive assessment of awareness, knowledge, and practices provides a holistic understanding of the intervention effects. Finally, the high program adherence and sustained improvement in practices at three months demonstrate the program’s effectiveness and potential for successful implementation.

## Conclusions

At baseline, intern dentists demonstrated insufficient awareness, knowledge, and practice related to e-waste management. The educational program was associated with improved knowledge and practices regarding e-waste management. Additionally, a positive correlation between knowledge and practices was established both pre- and post-intervention. While the pre-post design and the use of a single-institution sample preclude a definitive claim of causality, these findings serve as a promising proof-of-concept for the feasibility of such interventions. The modular nature of this program suggests high scalability for other dental curricula. These findings highlight the critical need for integrating e-waste education into dental programs curriculum to raise environmentally responsible practices among future dental professionals and ensure environmental sustainability.

## Supplementary Information

Below is the link to the electronic supplementary material.

Supplementary Material 1

Supplementary Material 2

Supplementary Material 3

## Data Availability

The datasets used and analyzed during the current study are available from the corresponding author upon reasonable request.

## Abbreviations

4R: Reuse, Recycle, Reduce, and Repurpose
EEE: Electrical and electronic equipment
e-waste: Electronic waste
IQR: Interquartile range
r_s_: Spearman coefficient test
SD: Standard deviation
WEEE: Waste electrical and electronic equipment
