# Supplementary material for: The impact of an educational program on the electronic waste management knowledge and practices of dental interns: an interventional study
Source: Sci Rep. 2026 Apr 13;16:12242. doi: 10.1038/s41598-026-46718-0 (PMC13076994; doi:10.1038/s41598-026-46718-0)
Supplement: Supplementary file 1 — Supplementary Material 1 [file 41598_2026_46718_MOESM1_ESM.docx]

**The impact of an educational program on the electronic waste management knowledge and practices of dental interns: An Interventional Study**

**Journal name:** Scientific Reports

**Authors:** Rana Samy Galal, Aleya Hanafy El-Zoka, Ebtisam Mohamed Fetohy, Mayada Mohamed Reda Moussa, Mohamed Fakhry Hussein

**Corresponding author:** Mohamed Fakhry Hussein: Department of Occupational Health and Industrial Medicine, High Institute of Public Health, Alexandria University, Alexandria, Egypt. Email: hiph-mohamedfakhry@alexu.edu.eg

**Supplementary Material I**

**Dear intern dentists,**

**Please take your time to answer this questionnaire to assess your awareness, knowledge, and practices regarding e-waste management. Put a mark in front of your answer.**

**Personal and socio-demographic Information:**

1. Sex:

(1) Male

(2) Female

2. Age: ..............

3. Marital status:

(1) Single

(2) Married

(3) Divorced

4. Number of family members: .............

5. Did you participate in any previous e-waste management programs?

(1) Yes

(0) No

6. What is your source of information?

(1) School

(2) University

(3) Friends and family

(4) Social media

(5) Television

(0) I have no information

**Questions related to awareness regarding E-waste management:**

7. Do you hear about e-waste/electronic waste?

(1) Yes

(0) No

8. Do you hear about E-Tadweer application?

(1) Yes

(0) No

9. Are you aware of E-waste recycling?

(1) Yes

(0) No

**Questions to assess knowledge regarding E-waste management:**

10. Unused electronics are waste

(1) Yes

(0) No

(0) Don’t know

11. E-waste causes negative health impacts on human health

(1) Yes

(0) No

(0) Don’t know

12. Improper E-waste disposal causes serious threats to environment

(1) Yes

(0) No

(0) Don’t know

13. E-waste causes air pollution

(1) Yes

(0) No

(0) Don’t know

14. Repair of broken electronic device is a way to reduce E-waste

(1) Yes

(0) No

(0) Don’t know

15. Are there any precious components in E-waste?

(1) Yes

(0) No

(0) Don’t know

16. Is it worth recovering and recycling E-waste materials?

(1) Yes

(0) No

(0) Don’t know

17. Are there any collection spots for E-waste in Egypt?

(1) Yes

(0) No

(0) Don’t know

18. Are there governmental policies/laws for e-waste management in Egypt?

(1) Yes

(0) No

(0) Don’t know

19. E-waste can dispose through 4R “Reduce, Reuse, Recycle, and Repurpose”

(1) Yes

(0) No

(0) Don’t know

20. Does E-waste recycling have any economic importance?

(1) Yes

(0) No

(0) Don’t know

21. Is formal recycling is an appropriate way of E-waste recycling?

(1) Yes

(0) No

(0) Don’t know

.22 What hazardous materials are in E-waste?

..............................................

**Practice of dentists regarding E-waste management:**

23. Current practice regarding electronics that are no longer in use

(0) Thrown in trash/landfills

(0) Burning/ incineration

(1) Kept at home

(2) Given to personal contact

(2) Exchanged with dealer

(3) Given to E-waste collector

24. Do you separate E-wastes from general waste

(2) Always

(1) Sometimes

(0) Never

25. Do you encourage others to recycle E-waste?

(2) Always

(1) Sometimes

(0) Never

26. Do you implement recycling practices for your family?

(2) Always

(1) Sometimes

(0) Never

27. Do you obtain eco-friendly products to reduce the impact of E-waste on environment?

(2) Always

(1) Sometimes

(0) Never

**Thanks a lot**
