# Supplementary material for: The impact of an educational program on the electronic waste management knowledge and practices of dental interns: an interventional study
Source: Sci Rep. 2026 Apr 13;16:12242. doi: 10.1038/s41598-026-46718-0 (PMC13076994; doi:10.1038/s41598-026-46718-0)
Supplement: Supplementary file 3 — Supplementary Material 3 [file 41598_2026_46718_MOESM3_ESM.docx]

**The impact of an educational program on the electronic waste management knowledge and practices of dental interns: An Interventional Study**

**Journal name:** Scientific Reports

**Authors:** Rana Samy Galal, Aleya Hanafy El-Zoka, Ebtisam Mohamed Fetohy, Mayada Mohamed Reda Moussa, Mohamed Fakhry Hussein

**Corresponding author:** Mohamed Fakhry Hussein: Department of Occupational Health and Industrial Medicine, High Institute of Public Health, Alexandria University, Alexandria, Egypt. Email: hiph-mohamedfakhry@alexu.edu.eg

Supplementary Material III

**Table (S1): Distribution of the studied samples according to awareness items (n = 76)**

| **Q** | **Awareness items** | **Pre-intervention** | | **Immediate post-intervention** | | **three months post-intervention** | | **Significance between periods** | | |
| --- | --- | --- | --- | --- | --- | --- | --- | --- | --- | --- |
|  |  | **No.** | **%** | **No.** | **%** | **No.** | **%** | **p_1_** | **p_2_** | **p_3_** |
| **1** | **Do you hear about E-waste/electronic waste?** |  |  |  |  |  |  |  |  |  |
|  | No | 67 | 88.2 | 0 | 0.0 | 0 | 0.0 | <0.001^*^ | <0.001^*^ | 1.000 |
|  | Yes | 9 | 11.8 | 76 | 100.0 | 76 | 100.0 |  |  |  |
| **2** | **Do you hear about E-Tadweer application?** |  |  |  |  |  |  |  |  |  |
|  | No | 76 | 100.0 | 2 | 2.6 | 6 | 7.9 | <0.001^*^ | <0.001^*^ | 0.572 |
|  | Yes | 0 | 0.0 | 74 | 97.4 | 70 | 92.1 |  |  |  |
| **3** | **Are you aware of e-waste recycling?** |  |  |  |  |  |  |  |  |  |
|  | No | 73 | 96.1 | 1 | 1.3 | 4 | 5.3 | <0.001^*^ | <0.001^*^ | 0.665 |
|  | Yes | 3 | 3.9 | 75 | 98.7 | 72 | 94.7 |  |  |  |

p1: p value for comparing between pre-intervention and immediate post-intervention

p2: p value for comparing between pre-intervention and three months post-intervention

p3: p value for comparing between immediate and three months post-intervention

*: Statistically significant at p ≤ 0.05

P value for Friedman test

**Table (S2): Distribution of the studied samples according to Knowledge items (n = 76)**

| **Q** | **Knowledge items** | **Pre-intervention** | | **Immediate post-intervention** | | **3 months post-intervention** | | **Significance between periods** | | |
| --- | --- | --- | --- | --- | --- | --- | --- | --- | --- | --- |
|  |  | **No.** | **%** | **No.** | **%** | **No.** | **%** | **p_1_** | **p_2_** | **p_3_** |
| **1** | **Unused electronics are waste** |  |  |  |  |  |  |  |  |  |
|  | No & Don’t know | 64 | 84.2 | 9 | 11.8 | 17 | 22.4 | <0.001^*^ | <0.001^*^ | 0.221 |
|  | Yes | 12 | 15.8 | 67 | 88.2 | 59 | 77.6 |  |  |  |
| **2** | **E-waste causes negative health impacts on human health** |  |  |  |  |  |  |  |  |  |
|  | No & Don’t know | 60 | 78.9 | 3 | 3.9 | 8 | 10.5 | <0.001^*^ | <0.001^*^ | 0.425 |
|  | Yes | 16 | 21.1 | 73 | 96.1 | 68 | 89.5 |  |  |  |
| **3** | **Improper E-waste disposal causes serious threats to environment** |  |  |  |  |  |  |  |  |  |
|  | No & Don’t know | 62 | 81.6 | 2 | 2.6 | 9 | 11.8 | <0.001^*^ | <0.001^*^ | 0.280 |
|  | Yes | 14 | 18.4 | 74 | 97.4 | 67 | 88.2 |  |  |  |
| **4** | **Electronic Waste causes air pollution** |  |  |  |  |  |  |  |  |  |
|  | No & Don’t know | 64 | 84.2 | 7 | 9.2 | 12 | 15.8 | <0.001^*^ | <0.001^*^ | 0.440 |
|  | Yes | 12 | 15.8 | 69 | 90.8 | 64 | 84.2 |  |  |  |
| **5** | **Repair of broken electronic device is a way to reduce e-waste** |  |  |  |  |  |  |  |  |  |
|  | No & Don’t know | 61 | 80.3 | 5 | 6.6 | 14 | 18.4 | <0.001^*^ | <0.001^*^ | 0.168 |
|  | Yes | 15 | 19.7 | 71 | 93.4 | 62 | 81.6 |  |  |  |
| **6** | **Are there any precious components in e-waste?** |  |  |  |  |  |  |  |  |  |
|  | No & Don’t know | 60 | 78.9 | 3 | 3.9 | 10 | 13.2 | <0.001^*^ | <0.001^*^ | 0.272 |
|  | Yes | 16 | 21.1 | 73 | 96.1 | 66 | 86.8 |  |  |  |
| **7** | **Is it worth recovering and recycling those materials?** |  |  |  |  |  |  |  |  |  |
|  | No & Don’t know | 60 | 78.9 | 4 | 5.3 | 12 | 15.8 | <0.001^*^ | <0.001^*^ | 0.182 |
|  | Yes | 16 | 21.1 | 72 | 94.7 | 64 | 84.2 |  |  |  |
| **8** | **Are there any collection spots for e-waste in Egypt?** |  |  |  |  |  |  |  |  |  |
|  | No & Don’t know | 66 | 86.8 | 3 | 3.9 | 6 | 7.9 | <0.001^*^ | <0.001^*^ | 0.649 |
|  | Yes | 10 | 13.2 | 73 | 96.1 | 70 | 92.1 |  |  |  |
| **9** | **Are there governmental policies/laws for e-waste management in Egypt?** |  |  |  |  |  |  |  |  |  |
|  | No & Don’t know | 66 | 86.8 | 8 | 10.5 | 19 | 25.0 | <0.001^*^ | <0.001^*^ | 0.082 |
|  | Yes | 10 | 13.2 | 68 | 89.5 | 57 | 75.0 |  |  |  |
| **10** | **E-waste can be disposed of through 3 R “Reduce, Reuse and Recycle"** |  |  |  |  |  |  |  |  |  |
|  | No & Don’t know | 67 | 88.2 | 5 | 6.6 | 15 | 19.7 | <0.001^*^ | <0.001^*^ | 0.132 |
|  | Yes | 9 | 11.8 | 71 | 93.4 | 61 | 80.3 |  |  |  |
| **11** | **Does e-waste recycling have any economic importance?** |  |  |  |  |  |  |  |  |  |
|  | No & Don’t know | 58 | 76.3 | 2 | 2.6 | 7 | 9.2 | <0.001^*^ | <0.001^*^ | 0.425 |
|  | Yes | 18 | 23.7 | 74 | 97.4 | 69 | 90.8 |  |  |  |
| **12** | **Is formal recycling an appropriate way of e-waste recycling?** |  |  |  |  |  |  |  |  |  |
|  | No & Don’t know | 61 | 80.3 | 11 | 14.5 | 18 | 23.7 | <0.001^*^ | <0.001^*^ | 0.248 |
|  | Yes | 15 | 19.7 | 65 | 85.5 | 58 | 76.3 |  |  |  |
| **13** | **What hazardous materials are in e-waste?** |  |  |  |  |  |  |  |  |  |
|  | 0 (wrong answer) | 72 | 94.7 | 7 | 9.2 | 22 | 28.9 | <0.001^*^ | <0.001^*^ | 0.0039^*^ |
|  | 1 (incomplete correct answer) | 2 | 2.6 | 50 | 65.8 | 40 | 52.6 |  |  |  |
|  | 2 (complete correct answer) | 2 | 2.6 | 19 | 25.0 | 14 | 18.4 |  |  |  |

P value for Friedman test

p1: p value for comparing between pre-intervention and immediate post-intervention

p2: p value for comparing between pre-intervention and three months post-intervention

p3: p value for comparing between immediate and three months post-intervention

*: Statistically significant at p ≤ 0.05

**Table (S3): Distribution of the studied samples according to practice items (n = 76)**

| **Q** | **Practice** | **Pre-intervention** | | **Immediate post-intervention** | | **3 months post-intervention** | | **Significance between periods** | | |
| --- | --- | --- | --- | --- | --- | --- | --- | --- | --- | --- |
|  |  | **No.** | **%** | **No.** | **%** | **No.** | **%** | **p_1_** | **p_2_** | **p_3_** |
| **1** | **Current practice regarding electronics that are no longer in use** |  |  |  |  |  |  |  |  |  |
|  | 1. Thrown in trash/landfills - Burning/ incineration | 13 | 17.1 | 7 | 9.2 | 4 | 5.3 | <0.001^*^ | <0.001^*^ | 0.224 |
|  | 1. Kept at home | 41 | 53.9 | 23 | 30.3 | 18 | 23.7 |  |  |  |
|  | 1. Given to personal contact - Exchanged with dealer | 17 | 22.4 | 11 | 14.5 | 16 | 21.1 |  |  |  |
|  | 1. Given to e-waste collector | 5 | 6.6 | 35 | 46.1 | 38 | 50.0 |  |  |  |
| **2** | **Do you separate electronic wastes from general waste?** |  |  |  |  |  |  |  |  |  |
|  | Never | 45 | 59.2 | 20 | 26.3 | 2 | 2.6 | 0.017^*^ | <0.001^*^ | <0.001^*^ |
|  | Sometimes | 22 | 28.9 | 46 | 60.5 | 24 | 31.6 |  |  |  |
|  | Always | 9 | 11.8 | 10 | 13.2 | 50 | 65.8 |  |  |  |
| **3** | **Do you encourage others to recycle e-waste?** |  |  |  |  |  |  |  |  |  |
|  | Never | 73 | 96.1 | 26 | 34.2 | 3 | 3.9 | <0.001^*^ | <0.001^*^ | <0.001^*^ |
|  | Sometimes | 1 | 1.3 | 41 | 53.9 | 49 | 64.5 |  |  |  |
|  | Always | 2 | 2.6 | 9 | 11.8 | 24 | 31.6 |  |  |  |
| **4** | **Do you implement recycling practices for you & your family?** |  |  |  |  |  |  |  |  |  |
|  | Never | 73 | 96.1 | 25 | 32.9 | 2 | 2.6 | <0.001^*^ | <0.001^*^ | <0.001^*^ |
|  | Sometimes | 1 | 1.3 | 41 | 53.9 | 48 | 63.2 |  |  |  |
|  | Always | 2 | 2.6 | 10 | 13.2 | 26 | 34.2 |  |  |  |
| **5** | **Do you obtain eco-friendly products to reduce the impact of e-waste on environment?** |  |  |  |  |  |  |  |  |  |
|  | Never | 69 | 90.8 | 16 | 21.1 | 1 | 1.3 | <0.001^*^ | <0.001^*^ | <0.001^*^ |
|  | Sometimes | 4 | 5.3 | 43 | 56.6 | 30 | 39.5 |  |  |  |
|  | Always | 3 | 3.9 | 17 | 22.4 | 45 | 59.2 |  |  |  |

p1: p value for comparing between pre-intervention and immediate post-intervention

p2: p value for comparing between pre-intervention and three months post-intervention

p3: p value for comparing between immediate and three months post-intervention

*: Statistically significant at p ≤ 0.05 p value for Friedman test

#: More than one answer

**
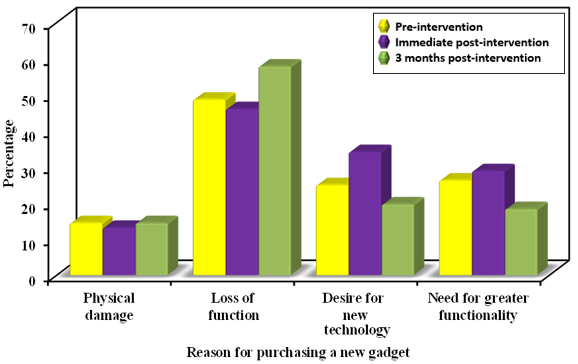
**

**Figure S1: Reasons for purchasing a new gadget among the studied intern dentists.**
